# Multimodal analgesia with parasternal plane block protocol within an enhanced recovery after cardiac surgery program decreases opioid use

**DOI:** 10.1016/j.xjon.2024.08.007

**Published:** 2024-09-07

**Authors:** Marc Darras, Clément Schneider, Sandrine Marguerite, Saadé Saadé, Anne-Lise Maechel, Walid Oulehri, Olivier Collange, Jean-Philippe Mazzucotelli, Paul-Michel Mertes, Michel Kindo

**Affiliations:** a Department of Cardiac Surgery, Hôpitaux Universitaires de Strasbourg, Nouvel Hôpital Civil, Strasbourg, France; b Department of Anesthesia and Intensive Care, Hôpitaux Universitaires de Strasbourg, Nouvel Hôpital Civil, Strasbourg, France

**Keywords:** cardiac surgery, multimodal analgesia, opioid, regional plane block, enhanced recovery after cardiac surgery, perioperative, care

## Abstract

**Objective:**

This study investigated the efficacy of a multimodal analgesia (MMA) with an opioid-sparing strategy, incorporating a parasternal plane block (PPB) within a systematic standardized Enhanced Recovery After Surgery (ERAS) program for patients undergoing elective cardiac surgery.

**Methods:**

From 2015 to 2021, 3153 patients underwent elective coronary artery bypass grafting and/or valve procedures. Patients were dichotomized by the presence or absence of an ERAS program including a perioperative MMA with an opioid-sparing approach and PPB protocols. Propensity score matching yielded 1026 well-matched pairs. The primary outcomes were the opioid-free rate and the opioid consumption in morphine milligram equivalents (MME) in the intensive care unit (ICU). The secondary outcomes were postoperative visual analog scale (VAS) scores, mechanical ventilation duration, ileus, delirium, bronchopneumonia, and length of ICU stay.

**Results:**

The ICU opioid-free rate was significantly increased in the ERAS group (94.0%) compared with the control group (19.9%; *P* < .001). The ERAS group had significantly lower opioid consumption in the ICU compared with the control group (median; 11.0 MME vs 31.0 MME; *P* < .001; respectively). The VAS scores were analogous between the control and ERAS groups during the ICU stay. In the ERAS group, mechanical ventilation duration, ileus, delirium, bronchopneumonia rates, as well as length of ICU stay, were significantly reduced (both *P* < .05).

**Conclusions:**

Within a systematic, standardized ERAS program, MMA with an opioid-sparing strategy and PPB enables opioid-free analgesia in the majority of patients, significantly decreases opioid consumption, and ensures effective postoperative pain management, thereby improving outcomes.

## Graphical abstract

**Figure:**
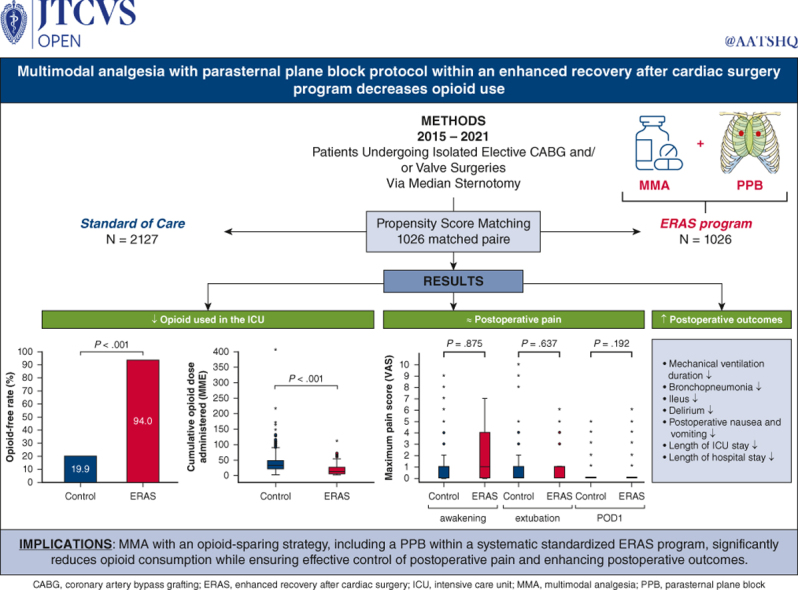

**Figure:**
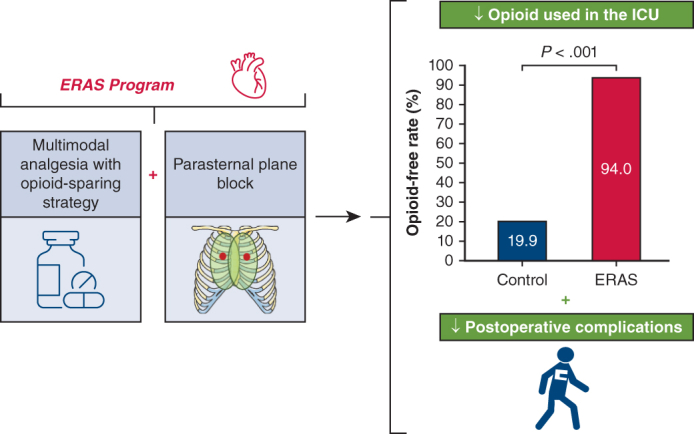
ERAS program with MMA and parasternal plane block.

**Central Message**

Implementing a multimodal analgesia protocol with an opioid-sparing strategy with parasternal plane blocks in an ERAS program reduces opioid use and improves postoperative recovery in cardiac surgery.

**Perspective**

In the ERAS perioperative program in cardiac surgery, the integration of multimodal analgesia, emphasizing an opioid-sparing strategy and regional blocks, stands as a critical advancement, substantially minimizing opioid requirements while maintaining effective pain management, leading to reduced opioid-associated adverse effects, improved postoperative outcomes, and decreased length of stay.

See Discussion on page 36.

The Enhanced Recovery After Surgery (ERAS) program in cardiac surgery incorporates perioperative protocols designed to enhance patient satisfaction, reduce postoperative morbidity, and shorten hospital stays.^1^^,^^2^ The ERAS program is becoming increasingly well-defined, with a growing body of evidence in the literature underscoring the benefits associated with such programs in cardiac surgery.^1^, ^2^, ^3^, ^4^, ^5^, ^6^, ^7^, ^8^

One of the key elements of the ERAS program is the implementation of multimodal analgesia (MMA) with an opioid-sparing strategy, aimed at mitigating the adverse effects associated with opioid use, enhancing postoperative pain management, and facilitating early rehabilitation.^1^^,^^2^^,^^9^, ^10^, ^11^, ^12^ In addition to the prescription of nonopioid analgesic drugs, the use of parasternal plane blocks (PPB) appears to be associated with a reduction in opioid consumption and an enhancement of postoperative pain control in cardiac surgery.^1^^,^^2^^,^^13^, ^14^, ^15^ In this study, we explored the impact of an MMA protocol combined with PPB within an ERAS program on patients undergoing cardiac surgery via median sternotomy.

## Methods

### Data Source

Perioperative and operative data were obtained from our database. This study protocol and publication of data were approved by the ethics committee of the French Society of Thoracic and Cardiovascular Surgery (CERC-SFCTCV-23140) on September 13, 2022. Written informed consent was obtained from all the study participants.

### Study Design

We conducted a retrospective analysis of patients who underwent elective coronary artery bypass grafting and/or valvular surgeries via a median sternotomy with cardiopulmonary bypass from January 2015 to December 2021. The cohort consisted exclusively of opioid-naïve individuals. Exclusion criteria included patients undergoing off-pump coronary artery bypass grafting, concomitant aortic procedures, emergent surgeries, and those requiring short-term circulatory or inotropic support. Pain medication administration records were reviewed, with all sources of opioid analgesia quantitatively standardized to morphine milligram equivalents (MME).^16^

**Figure 1:**
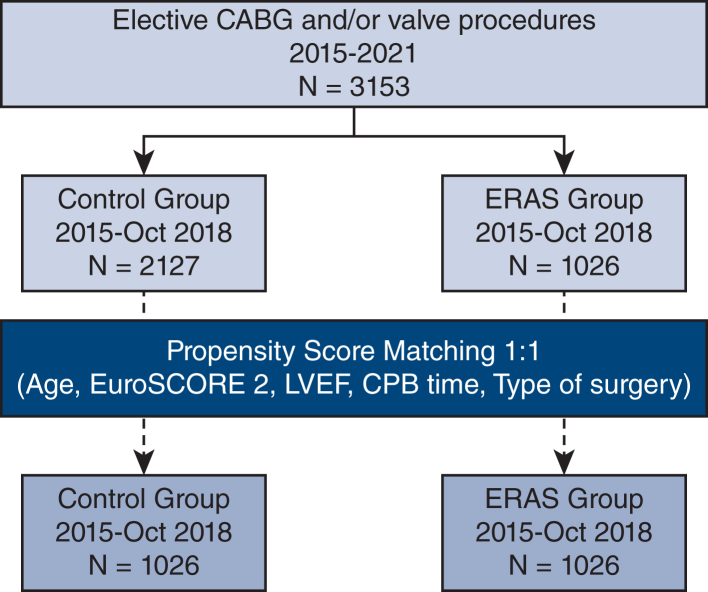
Patient flow diagram. *CABG*, Coronary artery bypass grafting; *ERAS*, Enhanced Recovery After Surgery; *EuroSCORE*, European System for Cardiac Operative Risk Evaluation; *LVEF*, left ventricular ejection fraction; *CPB*, cardiopulmonary bypass.

Patients were stratified according to the implementation date of our systematic, standardized ERAS program on November 1, 2018, creating 2 cohorts: a control group of 2127 patients who underwent surgery before this date and an ERAS group of 1026 patients who had surgery afterward. Figure 1 illustrates the study protocol through a flow diagram. Propensity score matching (PSM) resulted in 1026 patients in each group for subsequent analysis.

### ERAS Program

Our ERAS program has been previously described in detail.^3^, ^4^, ^5^ A summary of the nonanalgesic protocols included in our ERAS program is provided in Table E1.

Pain management in the control group was subject to the discretion of the medical staff, without a systematic and standardized protocol. In the immediate postoperative phase, the majority of patients in this group were administered either an intravenous (IV) patient-controlled analgesia system containing opioids and ketamine or a continuous opioid infusion pump, supplemented with opioid titration as required. Additional analgesics used included tramadol and nonopioid medications such as nefopam and nonsteroidal anti-inflammatory drugs (NSAIDs) with a nonselective cyclooxygenase inhibitor (ketoprofen), with their prescription contingent upon the physician's discretion, the level of pain control achieved in patients, and the presence of any potential contraindications.

In the ERAS group, perioperative analgesia protocols were standardized and systematically implemented from November 2018, alongside the implementation of other ERAS protocols (Tables 1 and E1). Patients were consistently informed and educated by both medical and nursing teams about perioperative pain management strategies. The anesthesiologist systematically performed an ultrasound-guided PPB before incision. The selection of the PPB was determined by the anesthesiologist and included either the bilateral ultrasound-guided transversus thoracic plane block (preferred technique for its extensive analgesic coverage), with the injection placed between the intercostal muscles and the transversus thoracis muscle, or the pectointercostal fascial plane block, with the injection positioned between the pectoralis major muscle and the intercostal muscles. The use of the pectointercostal fascial plane block was preferred in cases in which visualization of deep anatomical structures, particularly the internal thoracic artery, was difficult. During the first 24 hours, IV ketamine was administered (Table 1). Additional nonopioid analgesics employed included nefopam and NSAIDs (ketoprofen). Finally, additional analgesic measures were systematically implemented, such as early chest tube removal on the first postoperative day under light sedation or hypnosedation, early mobilization, and the routine use of a chest support belt.

**Table 1:** Perioperative analgesia in the ERAS group

Preoperative
Patient information and education on perioperative pain management Limiting preoperative sedative drugs: Preoperative hypnosedation was performed if necessary

Operative
Multimodal analgesia with opioid-sparing strategy: Intravenous sufentanil target controlled infusion: 0.1-0.5 ng/mL IV + Ketamine: 0.1 mg/kg/h IV + dexamethasone: 0.1 mg/kg IV bolus at incision + Magnesium: 3 g IV over 1 h at incision + Paracetamol: 1 g IV after induction + Nefopam: 40 mg IV over 1 h after induction Locoregional analgesia: An ultrasound-guided parasternal plane block was administered by the anesthesiologist at the fourth intercostal space, using either the transversus thoracic plane block (preferred choice) or the pectointercostal fascial plane block. Each technique involved a single injection of 20 mL of 0.325% ropivacaine on each side before the surgical incision.

Postoperative—ICU
Enhancement and continuation of information and education on postoperative pain management Multimodal analgesia with opioid-sparing strategy: Ketamine: 0.05-0.15 mg/kg/h during the first 24 h + Paracetamol: 1 g/6 h IV followed by PO + Nefopam: 20 mg IV/4 h for 2 d + MgSO4: 3 g IV/24 h during 2 d + Ketoprofen: 50 mg/6 h for 4 d if GFR ≥60 mL/min/1.73 m^2^ and/or operative and postoperative lactate level <3 mmol/L + Opioid: IV titration only if VAS score >3 Locoregional analgesia: Ultrasound-guided pectointercostal fascial plane block, if necessary, on the first postoperative day if VAS score >3 despite multimodal analgesia protocol Chest support belt Chest tube removal on the first postoperative day (if possible) under light sedation or hypnosedation

Postoperative—Department of Cardiac Surgery
Paracetamol: 1 g/6 h PO (systematic) + Nefopam: 20 mg/4 h PO + Ketoprofen: 100 mg Extended-Release/12 h during 2 d PO if GFR ≥60 mL/min/1.73 m^2^ + Opioid: 5 mg/4 h PO only if VAS score >3 (30 mg maximum/24 h) Chest support belt

*IV*, Intravenous; *ICU*, intensive care unit; *PO*, per os (by mouth); *GFR*, glomerular filtration rate; *VAS*, visual analog scale; *ERAS*, Enhanced Recovery After Cardiac Surgery.

### Study End Points

The primary outcomes were the opioid-free rate and the opioid consumption in MME during the entire intensive care unit (ICU) stay. The secondary outcomes were postoperative visual analog scale (VAS) scores, mechanical ventilation duration, bronchopneumonia, ileus, delirium, postoperative nausea and vomiting, renal function (glomerular filtration rate peak and Kidney Disease Improving Global Outcomes [KDIGO] classification), deep sternal wound infection, and length of stay. The definition of secondary criteria has been described elsewhere.^5^

### Statistical Analysis

**Figure E1:**
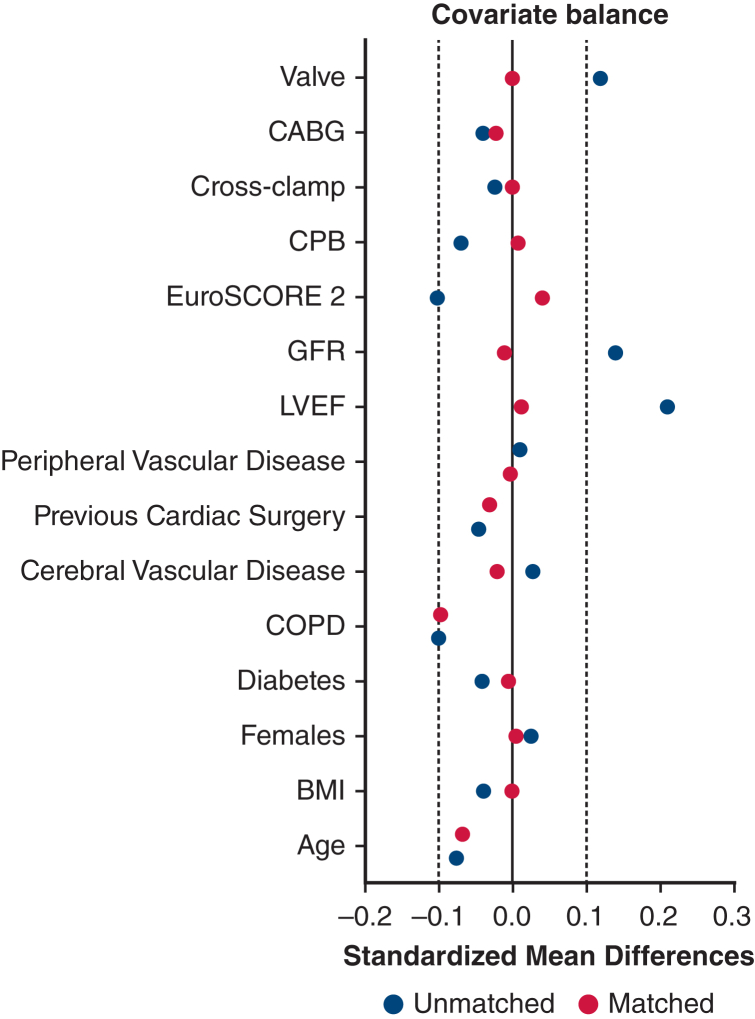
Standardized mean differences before and after propensity matching. *CABG*, Coronary artery bypass grafting; *CPB*, cardiopulmonary bypass; *EuroSCORE 2*, European System for Cardiac Operative Risk Evaluation 2; *GFR*, glomerular filtration rate, as determined with the Modification of Diet in Renal Disease study equation; *LVEF*, left ventricular ejection fraction; *COPD*, chronic obstructive pulmonary disease; *BMI*, body mass index.

Statistical analyses were conducted using R software (Version 2.15.3; R Foundation for Statistical Computing) and SPSS (Version 25.0; IBM Corp). Data are expressed as mean ± standard deviation for variables following a normal distribution and as median with interquartile range (IQR) for non-normally distributed variables. Normality of distribution was evaluated using skewness and kurtosis thresholds of 2, in addition to visual inspection of distribution plots. For categorical data, frequencies and percentages (n, %) were reported. χ^2^ or Fisher exact tests were applied to compare categorical variables, the latter being used when more than 20% of cells had expected counts less than 5. Continuous variables were compared using the Student *t* test or the Mann-Whitney *U* test, contingent upon normality. To mitigate confounding between control and ERAS cohorts, PSM was employed, incorporating variables such as age, left ventricular ejection fraction, European System for Cardiac Operative Risk Evaluation 2, cardiopulmonary bypass duration, and type of surgery. Logistic regression was used to calculate propensity scores, followed by 1:1 nearest-neighbor matching without replacement, adhering to a caliper width of 0.2 of the standard deviation of the logit-transformed propensity score. The matching process yielded well-balanced pairs of 1026 patients each, with absolute standardized mean differences less than 0.10 for all preoperative and operative variables. The balance achieved post-PSM is depicted in a Love plot (Figure E1). Given the absence of adjustment for multiple comparisons, *P* values should be interpreted as exploratory rather than confirmatory. Matched pairs were analyzed using the McNemar test for categorical variables and the Wilcoxon signed-rank test for continuous variables. All statistical tests were 2-sided.

## Results

### Preoperative and Operative Data

The preoperative and operative data are reported in Table 2 for the 2 groups before and after PSM. Detailed preoperative and operative data have been reported previously.^5^ Sternotomy closure was uniformly performed using traditional sternal wires in the 2 groups.

**Table 2:** Preoperative and operative characteristics

Variable	Entire population				Matched population			
	Control group n = 2127	ERAS group n = 1026	*P*	SMD	Control group n = 1026	ERAS group n = 1026	*P*	SMD
Age, y	68.2 ± 10.6	67.4 ± 10.4	.042	−0.076	68.1 ± 10.2	67.4 ± 10.4	.060	−0.067
Body mass index, kg/m^2^	27.9 ± 5.0	27.7 ± 5.3	.178	−0.039	27.7 ± 4.8	27.7 ± 5.3	.498	−0.001
Female	627 (29.5)	315 (30.7)	.517	0.006	286 (27.9)	315 (30.7)	.807	0.026
Diabetes	659 (31.0)	276 (26.9)	.019	−0.041	284 (27.7)	276 (26.9)	.000	−0.005
COPD	84 (3.9)	25 (2.4)	.029	−0.098	38 (3.7)	25 (2.4)	.085	−0.100
Cerebral vascular disease	138 (6.5)	62 (6.0)	.631	−0.020	54 (5.3)	62 (6.0)	.960	0.027
Previous cardiac surgery	113 (5.3)	51 (5.0)	.685	−0.031	56 (5.5)	51 (5.0)	.620	−0.047
Peripheral vascular disease	430 (20.2)	206 (20.1)	.928	0.011	214 (20.9)	206 (20.1)	.662	−0.002
Echocardiography								
LVEF, %	59.8 ± 9.8	61.8 ± 8.9	<.001	0.210	61.7 ± 8.6	61.8 ± 8.9	.737	0.011
Biology								
GFR, mL/min/1.73 m^2^	79.3 ± 24.7	82.8 ± 26.1	<.001	0.139	83.1 ± 23.3	82.8 ± 26.1	.921	−0.012
EuroSCORE 2, %	2.4 ± 3.1	2.1 ± 2.7	.082	−0.101	2.0 ± 2.2	2.1 ± 2.7	.482	0.041
Operative								
CPB time, min	111 (88-135)	110 (83-140)	.087	−0.069	108 (87-134)	110 (83-140)	.175	0.008
Crossclamp time, min	84 (66-108)	86 (62-112)	.528	−0.024	85 (65-109)	86 (62-112)	.117	0.001
CABG	1156 (53.3)	492 (48.0)	.001	−0.040	506 (49.3)	492 (48.0)	1.000	−0.022
Valve	1254 (59.0)	666 (64.9)	.009	0.119	659 (64.2)	666 (64.9)	1.000	0.001

Normally distributed data are expressed as the mean ± standard deviation, and non-normal data are expressed as the median (interquartile range [25-75th]). Categorical variables are represented as n (%). *P* value, control group versus ERAS group. *ERAS*, Enhanced Recovery After Cardiac Surgery; *SMD*, standardized mean difference; *COPD*, chronic obstructive pulmonary disease; *LVEF*, left ventricular ejection fraction; *GFR*, glomerular filtration rate, as determined with the Modification of Diet in Renal Disease study equation; *EuroSCORE 2*, European System for Cardiac Operative Risk Evaluation 2; *CPB*, cardiopulmonary bypass; *CABG*, coronary artery bypass grafting.

### Study End Points

Study end points for the matched population are reported in Table 3. All patients in the ERAS group received a PPB in the operating room, and no injuries to the internal thoracic artery were reported. On the first postoperative day in the ICU, 48 patients (4.7%) in the ERAS group required an ultrasound-guided pectointercostal fascial plane block because of significant pain (VAS score >3) despite our MMA protocol.

**Table 3:** Study end points in the matched population

Variable	Control group n = 1026	ERAS group n = 1026	*P*
Primary outcomes in the ICU			
Opioid-free	204 (19.9)	964 (94.0)	<.001
Opioid consumption, MME	31 (19-46)	11 (5-25)	<.001
Opioid consumption per hour, MME/h	0.6 (0.3-1.0)	0.2 (0.1-0.4)	<.001
Secondary outcomes			
Maximum pain score (VAS)			
Awakening	1 (0-1)	1 (0-5)	.875
Extubation	0 (0-1)	1 (0-1)	.637
First postoperative day	0 (0-0)	0 (0-1)	.192
Troponin I peak, μg/L	9.4 (5.4-17.0)	7.4 (4.3-12.8)	.692
Mechanical ventilation duration, h	6 (4-8)	4 (3-6)	<.001
Bronchopneumonia	139 (13.5)	92 (9.0)	.001
Ileus	45 (4.4)	27 (2.6)	.031
Delirium	54 (5.3)	27 (2.6)	.002
Postoperative nausea and vomiting	29 (2.8)	38 (3.7)	.264
Deep sternal wound infection	3 (0.3)	6 (0.6)	.507
GFR peak, mL/min/1.73 m^2^	79 (59-99)	83 (61-102)	.929
KDIGO stage 1 or greater	128 (12.5)	110 (10.7)	.218
Length of ICU stay, h	47 (41-72)	24 (21-74)	.008
Length of hospital stay, d	7 (6-8)	6 (5-7)	<.001

Normally distributed data are expressed as the mean ± standard deviation, and nonnormal data are expressed as the median (interquartile range [25-75th]). Categorical variables are represented as n (%). *P* value, control group versus ERAS group. *ERAS*, Enhanced Recovery After Cardiac Surgery; *ICU*, intensive care unit; *MME*, morphine milligram equivalents; *VAS*, visual analog scale; *GFR*, glomerular filtration rate, as determined with the Modification of Diet in Renal Disease study equation; *KDIGO*, Kidney Disease Improving Global Outcomes classification.

#### Opioid use

**Figure 2:**
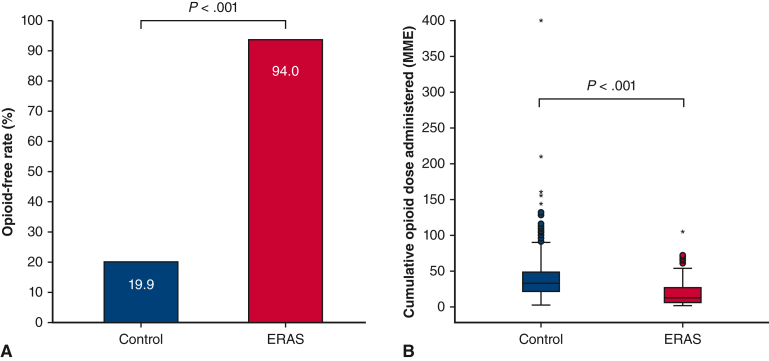
Opioid use in the ICU. Opioid-free rate in the ICU (A) and boxplot of distribution of cumulative opioid dose administration in the ICU (B). *Whiskers* represent ±1.5% of the interquartile range. Potential outliers are represented by *circles* or *stars* for control and ERAS groups, respectively. *ICU*, Intensive care unit; *ERAS*, Enhanced Recovery After Surgery; *MME*, morphine milligram equivalents.

In the ICU, the opioid-free rate significantly increased from 19.9% in the control group to 94.0% in the ERAS group (*P* < .001), representing an absolute risk reduction of opioid use of 74.1% (Table 3, Figure 2, *A*). The median cumulative opioid consumption in the ICU was 31 MME in the control versus 11 MME in the ERAS groups (Figure 2, *B*, *P* < .001). During the entire ICU stay, the median opioid consumption per hour was 0.6 MME/h in the control versus 0.2 MME/h in the ERAS groups (Table 3, *P* < .001). In the cardiac surgery unit, opioids were administered to 0.4% and 5.1% of the patients in the control and ERAS groups, respectively (*P* < .001). Among these patients receiving opioids, the median cumulative opioid consumption was 10 (IQR, 6-10) MME in the control and 5 (IQR, 5-10) MME in the ERAS groups (*P* = .901). During the entire hospital length of stay, the proportions of patients who were free of opioids remained consistent with those observed in the ICU for both groups. The median cumulative opioid consumption during the entire hospitalization period was 31 MME (IQR, 19-46) in the control group and 12 MME (IQR, 5-27) in the ERAS group (*P* < .001). Continuous delivery of opioids, either via IV patient-controlled analgesia or through IV opioid infusion pump, significantly decreased in the ERAS group compared with the control group (both *P* < .001, Table E1).

#### Other analgesic medications

Postoperative pain medications for the matched cohort are reported in Table E2. The use of ketamine in the ICU was significantly more prevalent in the ERAS group (*P* < .001). NSAID and nefopam administration rates significantly increased in the ERAS group (both *P* < .001). The use of tramadol within the ERAS group was significantly reduced (Table E2). No patients in either group were prescribed opioids upon discharge.

#### Pain score

**Figure 3:**
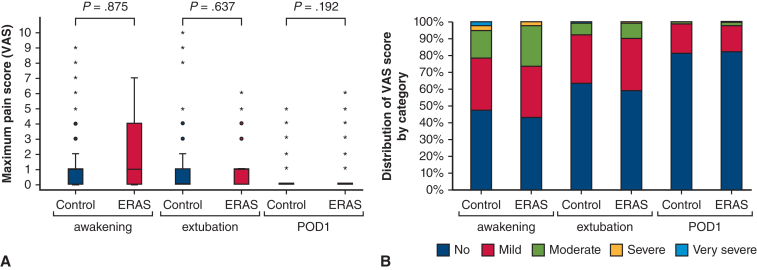
Pain score (VAS) in the ICU. Boxplot of distribution of maximal pain score (VAS) in the ICU and (A) distribution of VAS score by category (B). *Whiskers* represent ±1.5% of the interquartile range. Potential outliers are represented by *circles* or *asterisks* for control and ERAS groups, respectively. VAS score category: no pain (VAS = 0), minimal (VAS = 1-3), moderate (VAS = 4-5), severe (VAS = 6-7), very severe (VAS = 8-10). *VAS*, Visual analog scale; *ERAS*, Enhanced Recovery After Surgery; *POD*, postoperative day; *ICU*, intensive care unit.

The maximum pain scores assessed via the VAS did not demonstrate any statistically significant differences between the 2 groups in the matched cohort (Table 3, Figure 3, *A*). The distribution of VAS scores by category in the ICU revealed that the vast majority of patients experienced either no pain (VAS = 0), minimal pain (VAS = 1-3), or moderate pain (VAS = 4-5), whereas the occurrence of severe (VAS = 6-7) or very severe pain (VAS = 8-10) was exceptionally rare in both groups during the early postoperative period (Figure 3, *B*).

#### Postoperative morbidity

In the matched cohort, analysis of secondary outcomes revealed no significant differences between the control and ERAS groups in peak troponin I levels (*P* = .692), renal function impairment as indicated by glomerular filtration rate peak (*P* = .629), and KDIGO stage 1 or greater (*P* = .218) (Table 3). Within the entire matched cohort, 1041 patients (50.7%) received NSAIDs during the postoperative period. Compared with those who did not receive NSAIDs, patients treated with NSAIDs showed a decreased risk of postoperative acute renal failure (KDIGO stage 1 or greater: 6.6% vs 16.7%, *P* < .001) and lower troponin I peak levels (median 6.4; IQR, 3.6-11.3 vs median 8.8; IQR, 5.0-15.8, *P* < .001). The time to extubation was shorter in the ERAS group (*P* < .001), as were the lengths of ICU (*P* < .001) and hospital stays (*P* < .001) (Table 3). Fewer patients in the ERAS group experienced bronchopneumonia (*P* = .001), ileus (*P* = .031), and delirium (*P* = .002). There were no significant differences in the incidence of postoperative nausea and vomiting (*P* = .264) or deep sternal wound infections (*P* = .507).

## Discussion

Our findings demonstrate that an MMA protocol with an opioid-sparing strategy, coupled with a PPB within an ERAS program, results in effective postoperative pain management without the need of opioid use in most of the patients and with a significant reduction of opioid consumption. This protocol, in conjunction with other components of our ERAS program, enhances postoperative outcomes and improves the rehabilitation process for patients undergoing cardiac surgery via median sternotomy.

Effective postoperative pain management in cardiac surgery is crucial for minimizing stress, hemodynamic instability, and respiratory depression while maximizing patient comfort. Until recently, the standard analgesic treatment in cardiac surgery involved the use of opioids. However, the side effects of opioids present a significant limitation to their use.^9^^,^^16^ Consequently, the concept of MMA with an opioid-sparing strategy has evolved.^9^^,^^11^^,^^15^, ^16^, ^17^, ^18^ Therefore, recent ERAS guidelines advocate for the adoption of MMA protocols with an opioid-sparing strategy.^1^^,^^2^ Through the use of nonopioid drugs, similar to findings reported by other authors, we observed a significant reduction in postoperative opioid consumption while ensuring optimal pain control.^6^^,^^8^^,^^10^, ^11^, ^12^^,^^15^^,^^17^^,^^19^ It is crucial to emphasize that the low levels of opioid consumption and the high rate of opioid-free cases documented in our study are unprecedented in the medical literature.^6^^,^^8^^,^^10^, ^11^, ^12^^,^^15^^,^^17^^,^^19^ These outcomes can be attributed, in addition to the use of ketamine, to the frequent administration of NSAIDs both in the ICU and on the ward. NSAIDs have been clearly demonstrated to be effective in managing acute postoperative pain.^1^^,^^2^^,^^9^^,^^16^ However, until now, their use in the postoperative setting of cardiac surgery has been limited, primarily because of concerns about the risks of kidney injury, gastrointestinal complications, and thrombotic cardiovascular complications, especially with cyclooxygenase-2 inhibitors.^9^^,^^16^ With the prescribing criteria applied in our study, both within the ERAS group and in the overall matched population, the use of NSAIDs was not associated with an increased risk of acute renal failure and myocardial ischemia. Our findings, along with data from the literature, suggest that NSAIDs may be considered for selective use in patients undergoing cardiac surgery, as long as contraindications are strictly observed and the treatment duration is kept short.^2^

Another component of our MMA protocol was the incorporation of nefopam. Nefopam is a nonopioid, nonsteroidal, centrally acting analgesic drug. It has been demonstrated that the combined use of nefopam with other nonopioid drugs enhances pain relief and reduces opioid consumption.^20^ Our results advocate for this observation, demonstrating the avoidance of opioids in more than two-thirds of our patients, while maintaining optimal pain control. Furthermore, in the ERAS group, our MMA strategy was associated with a significant reduction in terms of mechanical ventilation duration, incidence of bronchopneumonia, ileus, delirium, and lengths of ICU and hospital stay. Cozowicz and colleagues^12^ also demonstrated that incorporating nonopioid analgesic drugs into opioid analgesia regimens was linked to a significant and stepwise decrease in adverse events.

Another key element in our postoperative pain management strategy within the ERAS group was the systematic implementation of a PPB before incision. The PPB provides effective analgesia in the operating room and throughout the immediate postoperative period, with a duration of 6 to 12 hours, thereby enabling a reduction in opioid consumption.^14^^,^^15^^,^^21^ On the basis of our experience, the administration of a PPB in the operating room is performed swiftly and has not been associated with any serious adverse events. Consequently, it is now considered the standard of care.

In our continuous effort to optimize pain management, we incorporated the administration during the surgery of dexamethasone and magnesium into our ERAS program, both of which have shown tangible benefits in reducing postoperative pain.^1^^,^^2^^,^^9^^,^^16^ It is important to note that within the ERAS group, we did not observe an increased risk of deep sternal wound infection despite the use of PPB, dexamethasone, or NSAIDs.

Finally, another crucial aspect of postoperative pain management is our management of chest tubes. Indeed, we implemented an early chest tube removal on the first postoperative day protocol within the ERAS group. We have demonstrated that this early removal can be safely executed in the majority of patients within the ERAS group.^5^ This removal was conducted under light sedation or hypnosedation with the aim of preventing any pain. Finally, all patients in the ERAS group were provided with a chest support belt for three weeks, primarily for analgesic purposes.

### Study Limitations

This study is subject to several limitations. First, as a single-center investigation, the findings may not be universally applicable. The specific type of PPB administered in the operating room for the ERAS group was not recorded in our database. Consequently, this study cannot compare the relative efficacy of the 2 different PPB techniques. It is important to note that the results observed in the ERAS group regarding opioid use and postoperative pain control cannot be attributed solely to the effects of the MMA and PPB protocols. Other components of our ERAS program, including patient education, the application of a postoperative chest support belt, early removal of chest tubes on the first postoperative day, and early mobilization, also likely contributed to these outcomes. In our study, the documentation of opioid-related adverse events was limited, capturing only the incidences of ileus, delirium, and postoperative nausea and vomiting. This limitation precludes a comprehensive analysis of the clinical implications of reduced opioid use. In addition, the absence of recorded total doses for nonopioid medications could potentially skew the outcomes. The study also grapples with the challenges posed by temporal variations and the learning curve associated with the ERAS implementation, although these were partially mitigated through the use of propensity score matching and the stability of the medical team. Despite these limitations, the study’s strengths lie in the meticulous application of a well-established, standardized ERAS program to a substantial and uniform patient cohort, bolstered by the institution's significant experience in integrating ERAS protocols within cardiac surgery.

## Conclusions

**Figure 4:**
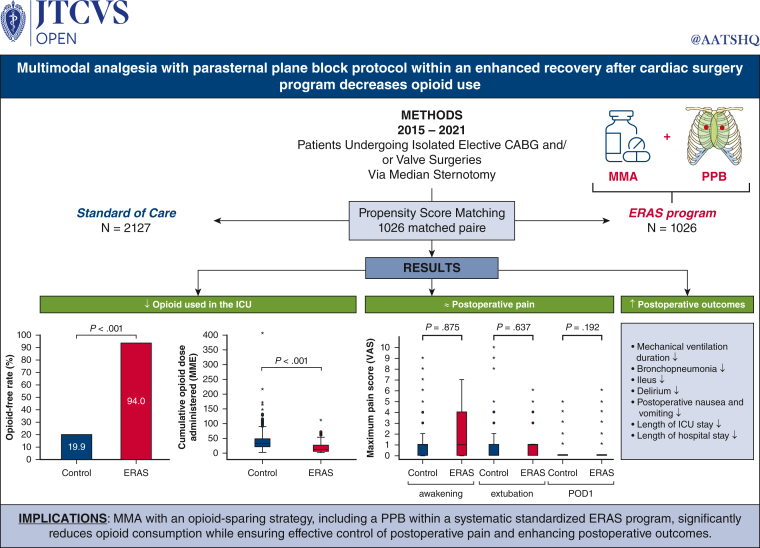
Effects of an MMA with opioid-sparing strategy with PPB protocol within an ERAS program for patients undergoing isolated CABG and/or valve surgery. Implementation of an MMA protocol with opioid-sparing strategy and PPB within an ERAS program significantly reduced opioid use and enhanced postoperative recovery in cardiac surgery patients. *CABG*, Coronary artery bypass grafting; *MMA*, multimodal analgesia; *PPB*, parasternal plane block; *ERAS*, Enhanced Recovery After Cardiac Surgery; *ICU*, intensive care unit; *VAS*, visual analog scale; *POD*, postoperative day; *RPB*, regional plane block.

Our study demonstrates that an MMA protocol with an opioid-sparing strategy, combined with a PPB within an ERAS program, allows efficient postoperative analgesia without the need for opioid use in the majority of patients, or with minimal opioid consumption (Figure 4). This analgesic strategy significantly reduces the incidence of opioid-related side effects and postoperative complications, thereby enhancing rehabilitation capabilities after cardiac surgery.

### Webcast

You can watch a Webcast of this AATS meeting presentation by going to: https://www.aats.org/resources/multimodal-analgesia-with-regi-7320.

**Figure:**
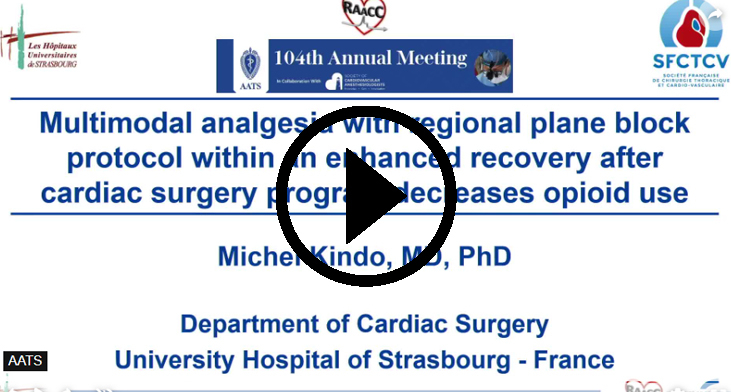

## Conflict of Interest Statement

The authors reported no conflicts of interest.

The *Journal* policy requires editors and reviewers to disclose conflicts of interest and to decline handling or reviewing manuscripts for which they may have a conflict of interest. The editors and reviewers of this article have no conflicts of interest.

## Figures and Tables

**Table E1:** Other systematic standardized protocols in the ERAS program

Preoperative protocols	• Patient information and education on the care pathway and systematic ERAS protocols • Alcohol and smoking cessation.∗ • Respiratory prehabilitation. • High-protein oral nutritional supplementation in the 8 d before the surgery. • Nasal *Staphylococcus aureus* screening and nasal decolonization if positive. • Oropharyngeal decontamination for 5 d. • Limitation of preoperative fasting. • Carbohydrate loading.
Operative protocols	• Protective lung ventilation. • Goal-directed therapy. • Transesophageal echocardiography.∗ • Optimized extracorporeal circulation. • Goal-directed transfusion.
Postoperative protocols	• Normothermia. • Half-seated position of 30° at admission in ICU. • Early tracheal extubation. • Postoperative nausea and vomiting prevention. • Early mobilization. • Early oral intake. • Early postoperative active and passive cardio-muscular and respiratory rehabilitation.

*ERAS*, Enhanced Recovery After Cardiac Surgery; *ICU*, intensive care unit.

∗ Protocol also present in the control group.

**Table E2:** Matched cohort pain medications during the postoperative course

Variable	Control group n = 1026	ERAS group n = 1026	*P*
ICU			
NSAID (ketoprofen)∗	332 (32.4)	499 (48.6)	<.001
Tramadol∗	185 (18.0)	4 (0.4)	<.001
Nefopam∗	462 (45.0)	974 (94.8)	<.001
Ketamine	730 (71.2)	907 (88.4)	<.001
IV-PCA (morphine + ketamine + droperidol)	536 (52.2)	8 (0.8)	<.001
IV opioid infusion pump	294 (28.7)	30 (2.9)	<.001
Cardiac surgery department			
NSAID (ketoprofen) PO	72 (7.0)	494 (48.1)	<.001
Tramadol PO	445 (43.4)	61 (5.9)	<.001
Nefopam PO	218 (21.2)	778 (78.8)	<.001
Opioid PO	4 (0.4)	52 (5.1)	<.001

Categorical variables are represented as n (%). *P* value, control group versus ERAS group. *ERAS*, Enhanced Recovery After Cardiac Surgery; *ICU*, intensive care unit; *NSAID*, nonsteroidal anti-inflammatory drug; *IV-PCA*, intravenous patient-controlled analgesia; *PO*, per os (by mouth).

∗ IV followed by oral administration.
